# Integrated assessment of selectivity, soil behavior, and biochar-mediated release for the botanical herbicide precursor S-(-)-Spirobrassinin

**DOI:** 10.3389/fpls.2026.1787286

**Published:** 2026-03-06

**Authors:** Yu Wang, Dong Wang, Qian Zhang, Meng Zhang, Ruixin Yang, Weiqi Li, Kaixuan Li, Jianxiu Hao, Hongyou Zhou

**Affiliations:** 1 College of Horticulture and Plant Protection, Inner Mongolia AgriculturalUniversity, Hohhot, Inner Mongolia Autonomous Region, China; 2 Bayannur Cultivated Land Quality Monitoring and Protection Center,Bayannur, Inner Mongolia Autonomous Region, China; 3 Bayannur Modern Agriculture and Animal Husbandry Development Center,Bayannur, Inner Mongolia Autonomous Region, China; 4 Hulunbuir Agricultural Technology Extension Center, Hulunbeir,Inner Mongolia Autonomous Region, China; 5 Tongliao City Horqin District Agricultural Technology Extension Center,Tongliao, Inner Mongolia Autonomous Region, China

**Keywords:** biochar, degradation behavior, microbial diversity, molecular dynamics simulation, S-(-)-Spirobrassinin, safety, target weeds

## Abstract

S-(-)-Spirobrassinin is a secondary metabolite derived from broccoli, exhibiting significant weed-suppressive activity and serving as a promising lead compound for botanical herbicide development. However, its practical application is constrained by unanswered questions regarding crop selectivity, environmental fate, and particularly its rapid soil degradation. To address these gaps, this study employed a combination of bioassays, soil analytics, and molecular dynamics simulations to comprehensively evaluate its herbicide potential and explore a biochar-based carrier strategy for sustained release. Our results demonstrated that S-(-)-Spirobrassinin exhibited differential tolerance on *Setaria italica* compared to *Sorghum bicolor*, *Avena sativa* and *Brassica napus*, while demonstrating superior efficacy against graminaceous weeds over broadleaf weeds, indicating a notable degree of selectivity. Soil incubation experiments revealed that the compound degraded rapidly, with only 2.7 μg/mL detectable after 21 days, and induced transient shifts in microbial community structure, reducing overall diversity and altering the relative abundance of key phyla such as Proteobacteria and Actinobacteria. To overcome the degradation limitation, molecular simulations revealed that S-(-)-Spirobrassinin can be strongly adsorbed onto carbonaceous surfaces and confined within micropores, underpinning the theoretical basis for using biochar as an effective controlled-release carrier. This work not only elucidates the crop selectivity and soil behavior of S-(-)-Spirobrassinin but also proposes a mechanism informed strategy to enhance its persistence, providing a holistic foundation for developing this natural product into an eco-friendly smart herbicide system.

## 1 Introduction

S-(-)-Spirobrassinin, an indole alkaloid secondary metabolite derived from broccoli, has emerged as a potent botanical herbicide lead due to its strong inhibitory effects on weed germination and growth (Wang et al., 2023). To translate this promising activity into a practical agrochemical, however, two intertwined barriers must be overcome: first, its ecological safety profile, encompassing crop selectivity and impact on soil microbial communities, remains largely uncharacterized; second, its potential for rapid environmental degradation threatens field efficacy and necessitates the development of a formulation strategy to enhance persistence. Controlled release using a carrier matrix represents a viable approach to mitigate rapid degradation. Biochar, a carbon-rich porous material, has garnered attention as a sustainable pesticide carrier owing to its high adsorption capacity and tunable physicochemical properties. Nevertheless, the rational design of biochar for targeted delivery of S-(-)-Spirobrassinin is hampered by a fundamental knowledge gap: a lack of molecular-level understanding of their interactions, adsorption configurations, and diffusion dynamics within biochar nanopores.

Currently, the overuse of chemical pesticides has significantly impacted agricultural ecosystems. Interestingly, some studies have suggested that synthetic herbicides may affect non-target organisms by altering soil microbial communities and their functions (Daisley et al., 2022; Akhter et al., 2024). Given the crucial role of soil microorganisms in agricultural ecosystems, understanding the changes S-(-)-Spirobrassinin induces in microbial communities is of great importance. Natural products can be decomposed and utilized by soil microorganisms as carbon or nitrogen sources (Scavo et al., 2019). Biochar, as a soil amendment and slow-release carrier, plays a significant regulatory role in the structure and function of soil microbial communities. Studies have shown that applying corn biochar to Mediterranean vineyard soils significantly increased soil organic carbon content and water retention capacity. However, short-term microbial responses were more complex, with biochar potentially suppressing total microbial biomass while showing no significant effects on microbial diversity or soil invertebrates. These findings indicate that the impact of biochar on soil microecology is complex and varies both spatially and temporally (Andrés et al., 2019). The combined application of corn biochar and functional microorganisms has been shown to significantly enhance soil enzyme activity and microbial diversity, thereby facilitating the stabilization and ecological remediation of soil pollutants (Tu et al., 2020). Further research has confirmed that surface-modified biochar, when applied in synergy with microorganisms, can effectively improve soil nutrient use efficiency and increase the complexity of microbial ecological networks (Cheng et al., 2025). However, research on the microbial regulatory mechanisms, long-term effects, and interactions of biochar with controlled-release herbicides still lacks systematic field data and mechanistic explanations.

In recent years, the development of slow-release herbicide carrier materials were widely applied in the field of pesticide science. Biodegradable polymers such as polyhydroxybutyrate and porous materials such as biochar and silica-based materials were widely applied in the construction of slow-release herbicide systems due to their favorable controlled-release properties and environmental friendliness. Cao et al. employed polyhydroxybutyrate microcapsules to encapsulate trifluralin, which significantly improved the herbicide’s photostability and field persistence. They optimized particle size distribution and encapsulation efficiency, achieving precise control over the slow-release effect by adjusting preparation parameters (Cao et al., 2019). Forini et al. developed magnetically responsive polycaprolactone microsphere carriers, enabling the controlled release and environmental recoverability of atrazine, thereby reducing toxicity risks to non-target plants (Forini et al., 2020). In the field of biochar, Liang et al. incorporated bamboo biochar into a soybean protein hydrogel system, which enhanced the material’s moisture retention and slow-release fertilizer efficiency, promoted crop growth, and enriched the soil microbial community (Liang et al., 2024). However, current research predominantly focuses on the macroscopic characterization of adsorption performance, while the molecular-scale interaction patterns, dynamic adsorption-release processes, and structure-activity relationships between S-(-)-Spirobrassinin and biochar remain unexplored. This knowledge gap limits the theoretical guidance for designing biochar carriers, hindering the achievement of precise controlled release. Therefore, it is imperative to employ molecular dynamics simulations as a tool to elucidate the binding mechanisms, diffusion behaviors, and energy changes between the two at the atomic level.

It is well established that biochar can effectively immobilize organic pollutants through various mechanisms such as physical adsorption, electrostatic interactions, π–π stacking, and hydrogen bonding, demonstrating significant promise in soil amendment, carbon sequestration, and pollution remediation (Dong et al., 2024; Ahmed et al., 2018). However, current research has largely focused on the adsorption capacity and macroscopic kinetics of biochar, while the microscopic adsorption–diffusion mechanisms, molecular-scale binding configurations, and the structure–activity relationships governing their slow-release performance remain insufficiently elucidated. This fundamental gap hinders the rational design and performance optimization of biochar-based slow-release materials.

This work seeks to provide a comprehensive foundation for developing S-(-)-Spirobrassinin into an environmentally friendly smart herbicide. Our investigation encompasses four specific aims: (i) to evaluate selective phytotoxicity by quantifying differential effects on crops and weeds, thereby defining its selectivity window; (ii) to characterize environmental behavior through soil degradation kinetics and analysis of short-term impacts on soil microbial community structure and diversity; (iii) to elucidate the molecular adsorption mechanism by employing molecular dynamics simulations to analyze adsorption configurations and multi-stage kinetics on a carbon model; and (iv) to assess carrier feasibility by integrating simulation-derived structure-activity relationships to quantitatively evaluate and guide the design of carbon-based controlled-release systems.

## 2 Materials and methods

### 2.1 Materials

The target compound was S-(-)-Spirobrassinin (C_11_H_10_N_2_OS_2_), with a molecular weight of 250.34 g·mol^-^¹. The soil sample was collected from an experimental field in Hohhot, Inner Mongolia autonomous region(44°08′ N, 111°41′ E), where the previous crop had been oats. During sampling, the surface soil was removed and soil from the 5–10 cm layer was taken using the five-point sampling method, followed by storage at -80 °C. Seeds were provided by the Inner Mongolia Autonomous Region Key Laboratory of Biopesticide Creation and Resource Utilization at Inner Mongolia Agricultural University. Corn stover biochar was purchased from Zhengzhou, Henan Province, China.

### 2.2 Methods

#### 2.2.1 Seed germination and selectivity assessment

The selected crops included *Sorghum bicolor*, *Avena sativa*, *Setaria italica*, and *Brassica napus*. The weeds included *Convolvulus arvensis*, *Amaranthus retroflexus*, *Panicum miliaceum*, *Echinochloa crus-galli*, and *Setaria viridis*. Uniformly sized seeds were surface-sterilized by soaking in a 0.2% sodium hypochlorite solution for 10 minutes, followed by three rinses with distilled water. Two layers of sterile filter paper were placed at the bottom of a 60 mm diameter Petri dish, and 10 seeds were evenly distributed in each dish. S-(-)-Spirobrassinin was dissolved in distilled water to a concentration of 0.25 mg/mL. Then, 2 mL of the solution was added to each Petri dish, with three replicates per treatment. An equal volume of distilled water was added to the control dishes. The germination and growth assay was conducted in an artificial climate incubator under the following conditions: temperature at 25 ± 0.5°C, humidity at 85%, and a 12 h light/12 h dark photoperiod. After 7 days, the root and shoot lengths were measured.

#### 2.2.2 Dose response bioassay and IC_50_ determination

An aqueous solution of S-(-)-Spirobrassinin was prepared at five concentrations (0.01, 0.1, 0.15, 0.2, and 0.25 mg/mL). Tribenuron-methyl was used as a positive herbicide control, and distilled water served as the blank control. A filter paper-based Petri dish assay was used for seed germination. Uniformly germinated seeds of *P. miliaceum* and *A. retroflexus* were selected, and ten seeds were placed in each dish. The incubation conditions were identical to those described in section 2.2.1. Each treatment was set up with three biological replicates, with ten seeds per replicate. After 7 days of treatment, the shoot/stem length and root length of the seedlings were measured. The inhibition rate was calculated based on growth reduction relative to the blank control. A linear regression between concentration and inhibition rate was performed using GraphPad Prism 8, from which the half-maximal inhibitory concentration (IC_50_) was derived.

#### 2.2.3 Experiment on the effects of S-(-)-Spirobrassinin on soil microorganisms

To investigate the potential regulatory effect of biochar on the environmental behavior of S-(-)-Spirobrassinin, four treatment groups were established: Control, biochar, biochar + S-(-)-Spirobrassinin, and S-(-)-Spirobrassinin alone. Each treatment was performed in triplicate. S-(-)-Spirobrassinin was dissolved in water with heating to prepare a 0.25 mg·mL^-^¹ stock solution. For the S-(-)-Spirobrassinin treatment, 2 mL of the prepared S-(-)-Spirobrassinin solution was added to 2 g of soil. For the biochar+S-(-)-Spirobrassinin treatment, 0.5 g of biochar and 2 mL of the S-(-)-Spirobrassinin solution were added to 2 g of soil. For the biochar treatment, 0.5 g of biochar was added to 2 g of soil. The control group consisted of untreated soil. For all groups, the soil and additives were thoroughly mixed and then incubated statically in 1 mL centrifuge tubes.

#### 2.2.4 Soil DNA extraction

After 14 days, 1g soil samples were collected from the four treatment groups and the control group. The total genomic DNA was extracted from these samples using the OMEGA Soil DNA Kit, followed by storage at -20 °C.

#### 2.2.5 PCR amplification

The V3-V4 region of the bacterial *16S rRNA* gene was amplified by PCR using the forward primer 338F (5’-ACTCCTACGGGAGGCAGCA-3’) and the reverse primer 806R (5’-GGACTACHVGGGTWTCTAAT-3’). The *ITS1* region of the fungal *ITS* gene was amplified using the forward primer ITS1F (GGAAGTAAAAGTCGTAACAAGG) and the reverse primer ITS2 (GCTGCGTTCTTCATCGATGC). The PCR reaction mixture (25 μL total volume) consisted of 5 μL of 5× reaction buffer, 0.25 μL of Fast pfu DNA Polymerase (5 U/μL), 2 μL of dNTPs (2.5 mM each), 1 μL of each primer (10 μM), 1 μL of DNA template, and 14.75 μL of ddH_2_O. The thermal cycling conditions were as follows: initial denaturation at 98 °C for 5 min; followed by 25 cycles of denaturation at 98 °C for 30 s, annealing at 53 °C for 30 s, and extension at 72 °C for 45 s; with a final extension at 72 °C for 5 min. The PCR amplicons were purified using Vazyme DNA Clean Beads, and their concentration was quantified using the Quant-iT PicoGreen dsDNA Assay Kit. Following quantification, equimolar amounts of the amplicons were pooled. Paired-end sequencing was then performed using the MiSeq Reagent Kit v3.

#### 2.2.6 Sequencing data processing

Microbiome bioinformatics analysis was performed using QIIME2 version 2019.4. Briefly, raw sequence data were first demultiplexed using the demux plugin. Primers were then trimmed using the cutadapt plugin. Subsequently, sequences underwent quality filtering, denoising, merging, and chimera removal via the DADA2 plugin. The resulting non-singleton amplicon sequence variants (ASVs) were aligned with MAFFT and used to construct a phylogenetic tree with FastTree2.

#### 2.2.7 Analysis of soil microbial community diversity

Sequence data analysis was primarily conducted using QIIME2 and R packages v3.2.0. Alpha diversity indices, including the Shannon and Simpson indices at the ASV level, were calculated from the ASV table in QIIME2 and visualized as box plots. Rank abundance curves at the ASV level were generated to compare the richness and evenness of ASVs across samples. Beta diversity analysis was performed using the Jaccard, Bray-Curtis, and UniFrac distance metrics to investigate structural changes in the microbial communities. The results were visualized through principal coordinate analysis (PCoA).

#### 2.2.8 Detection of S-(-)-Spirobrassinin degradation period

The chromatographic analysis was performed using an Agilent SB-C18 column (100 mm × 2.1 mm,1.7 μm) maintained at 40 °C, with a mobile phase consisting of 5 mmol/L acetonitrile (A)and water (B) at a flow rate of 0.35 mL/min and an injection volume of 5 μL. The gradient elution program started with 5% B, increased linearly to 95% B over 9.00 min, held for 1 min, returned to 5% B between 10.00 and 11.10 min, and re-equilibrated until 14.00 min. Detection was conducted via electrospray ionization in positive mode with multiple reaction monitoring, with the ion spray voltage set at 5500 V, the ion source temperature at 350 °C, the collision gas pressure at 379 kPa, the curtain gas pressure at 241 kPa, and the auxiliary gas pressure at 414 kPa. The method detection limit and quantification limit were determined based on signal-to-noise ratios of S/N = 3 and S/N = 10 for the quantitative ion chromatographic peak, respectively. Specific data regarding the detection limits, recovery rates, and other relevant parameters are provided in **Supplementary Table 3**. Standard solutions of S-(-)-Spirobrassinin were prepared at concentrations of 0.55, 1.1, 5.5, 11, 27.5, and 55 ng/μL. A standard curve was plotted with concentration as the x-axis and peak area as the y-axis. After adding a 2 mg S-(-)-Spirobrassinin solution to the soil, the samples were stored at room temperature. The compound was extracted from the soil using chromatographic-grade methanol at intervals of 7, 14, 21, 25, and 28 days. Following each extraction, the samples were centrifuged at 5000 rpm for 15 minutes, and the supernatant was collected. This methanol extraction process was repeated three times, and the supernatants were combined. The combined supernatant was then evaporated using a rotary evaporator to obtain the total extracted residue. The mass of the residue was recorded. The resulting compound was dissolved in 1 mL of chromatographic methanol, filtered through a 0.22 μm organic filter membrane, and transferred into a vial for liquid chromatography analysis.

#### 2.2.9 Biochar model construction

A graphite model was built using Materials Studio software. The model employed graphite sheet units randomly assembled into an amorphous nanoporous carbon structure with a specific density to approximate the complex structure of real biochar. This nanoporous carbon model was used for computational simulations. To better mimic the intricate internal structure of biochar, where graphite sheet units of varying sizes are interwoven, a nanoporous carbon model with a density of 0.5 g/cm³ was constructed using seven-ring graphite sheet units (Wolak and Orzechowska-Zięba, 2024; Wood et al., 2024). The structure file of S-(-)-Spirobrassinin was obtained from the PubChem database (https://pubchem.ncbi.nlm.nih.gov/compound/188830). The molecule was subjected to preliminary energy minimization to eliminate unreasonable bond angles and lengths, resulting in a stable initial structure.

#### 2.2.10 Simulation methods

The adsorption of S-(-)-Spirobrassinin by biochar was simulated using the metropolis method of grand canonical monte carlo (GCMC) within the sorption module of materials studio. The adsorption heat, adsorption amount, and adsorption isotherms of S-(-)-Spirobrassinin were obtained under temperature: 290 K and pressure: 0.1 MPa. After obtaining the initial adsorption configuration, structural optimization was performed to achieve the most stable adsorption configuration with the lowest energy. Considering the characteristics of the adsorption system, the COMPASS II force field was selected to ensure accurate parameter matching for carbon atoms in graphite and all atoms in S-(-)-Spirobrassinin.

The boundary conditions were set as periodic in the x- and y-directions and non-periodic in the z-direction, with a vacuum layer included. The simulation time step was set to 1 fs, with a total simulation duration of ≥100 ps; the GCMC simulation comprised ≥1×10^6^ steps to ensure statistical reliability (Barzegar and Feyzi, 2025). The steepest descent method was first applied, iterating until energy convergence (convergence criterion set to 1×10^-4^ kcal/mol), to eliminate initial system stress and obtain a stable, energy-minimized initial configuration, providing a foundation for subsequent simulations. Based on the energy-minimized model, the simulation was run while monitoring the fluctuation of system energy in real time. When the energy fluctuation stabilized within ±5%, the system was considered to have reached equilibrium. Trajectory files from the equilibrium stage were then extracted for the calculation of radial distribution function (RDF) and mean square displacement(MSD).

For the “biochar–S-(-)-Spirobrassinin molecule” system, GCMC simulations were run to calculate the adsorption amount of S-(-)-Spirobrassinin on the graphite surface. Energy changes during adsorption were recorded to provide data for calculating adsorption heat. Key simulation data were extracted using the “Analysis” module in Materials Studio. “Adsorption amount–pressure” data were exported from GCMC results to generate the Sorption Loading curve. Based on the relationship between adsorption amount and temperature, the isosteric heat of adsorption was calculated using the Clausius–Clapeyron equation, yielding average, minimum, and maximum adsorption heat data. The radial distribution function was calculated from the equilibrium simulation trajectory to analyze the short-range interactions between S-(-)-Spirobrassinin and atoms on the biochar surface. The mean square displacement of S-(-)-Spirobrassinin was calculated based on the simulation trajectory to evaluate its diffusion capability on the biochar surface. The Atom Volumes Field tool was used to calculate the occupied volume, free volume, and specific surface area(SSA) of the system, analyzing the spatial structural characteristics of the graphite substrate.

#### 2.2.11 Data analysis

Statistical analysis was performed using the F-test or Levene’s test, and statistical significance was assessed using two-tailed t-tests. Data are presented as mean ± standard error. A *p*-value < 0.05 was considered statistically significant between groups, denoted as *p < 0.05, **p < 0.01, ***p < 0.001.

## 3 Results

### 3.1 Selectivity screening of S-(-)-Spirobrassinin on weeds and crops

**Figure 1:**
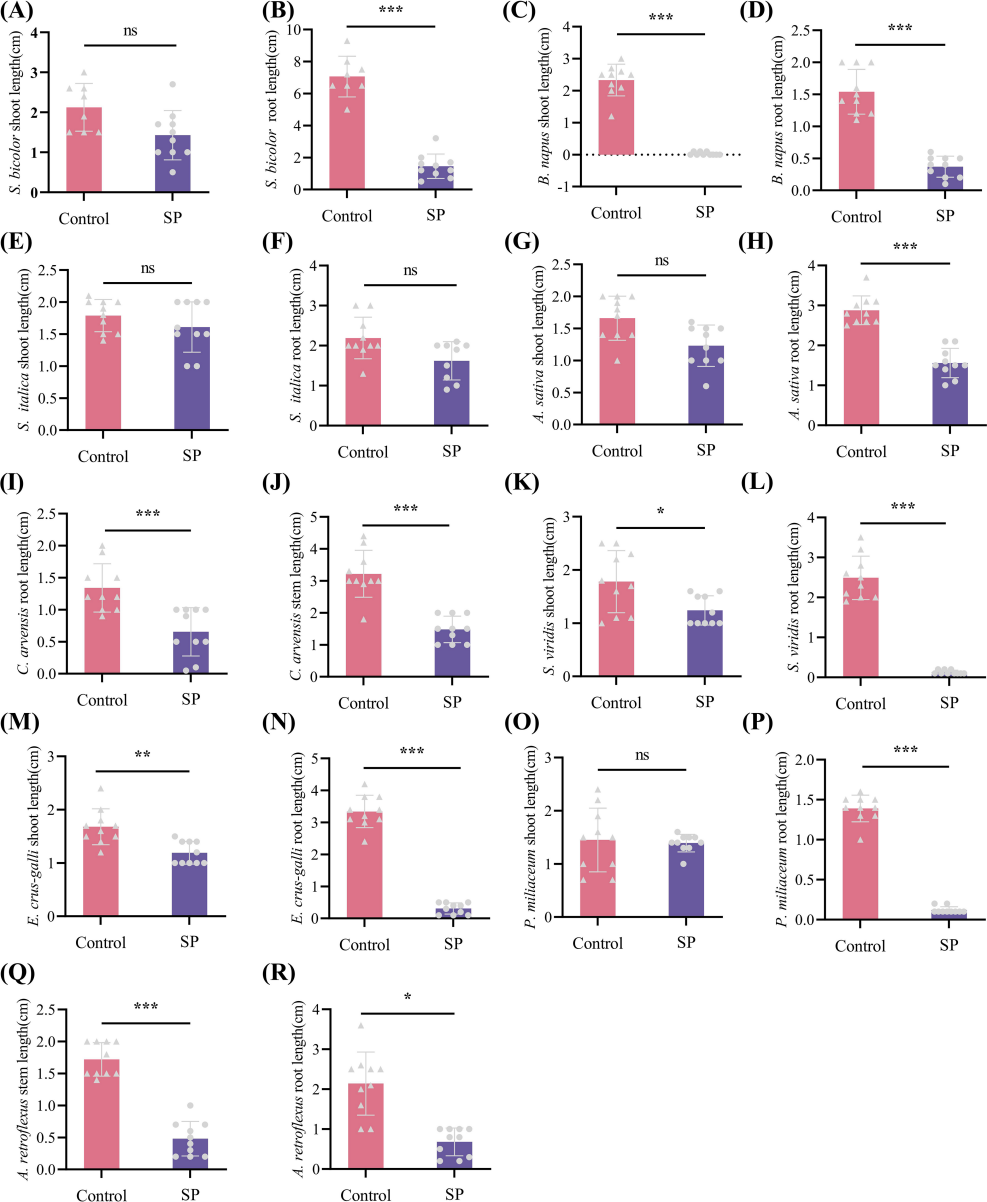
Selectivity of S-(-)-Spirobrassinin towards different crops and weeds. *p<0.05, **p<0.01, ***p<0.001; 'ns' indicates no significant difference after t-test; each bar represents the mean ± standard error; different symbols within columns denote different treatments. The untreated group is referred to as control, and the group treated with S-(-)-Spirobrassinin is abbreviated as SP. **(A)** Shoot length of *S. bicolor*, **(B)** Root length of *S. bicolor*, **(C)** Shoot length of *B. napus*, **(D)** Root length of *B. napus*, **(E)** Shoot length of *S. italica*, **(F)** Root length of *S. italica*, **(G)** Shoot length of *A. sativa*, **(H)** Root length of *A. sativa*, **(I)** Root length of *C. arvensis*, **(J)** Stem length of *C. arvensis*, **(K)** Shoot length of *S. viridis*, **(L)** Root length of *S. viridis*, **(M)** Shoot length of *E. crus-galli*, **(N)** Root length of *E. crus-galli*, **(O)** Shoot length of *P. miliaceum*, **(P)** Root length of *P. miliaceum*, **(Q)** Stem length of *A. retroflexus*, and **(R)** Root length of *A. retroflexus*.

To evaluate the selectivity of S-(-)-Spirobrassinin, four crops (*Sorghum bicolor*, *Avena sativa*, *Setaria italica*, and *Brassica napus*) and five weed species (*Convolvulus arvensis*, *Amaranthus retroflexus*, *Panicum miliaceum*, *Echinochloa crus-galli*, and *Setaria viridis*) were screened using the Petri dish germination assay. For the crops, the root lengths of *S. bicolor*, *A. sativa* and *B. napus* seedlings were significantly inhibited. In contrast, *S. italica* seedlings were not significantly affected, indicating that *S. italica* exhibited tolerance to S-(-)-Spirobrassinin. For the weeds, root elongation in all five weed seedlings was inhibited by S-(-)-Spirobrassinin. The inhibitory effect was stronger on the graminaceous weeds (*P. miliaceum*, *E. crus-galli and S. viridis*) than on the broadleaf weeds (*C. arvensis* and *A. retroflexus*), demonstrating that S-(-)-Spirobrassinin exhibits superior herbicidal activity against graminaceous weeds compared to broadleaf weeds (**Figure 1**).

### 3.2 Dose dependent herbicidal activity of S-(-)-Spirobrassinin

**Figure 2:**
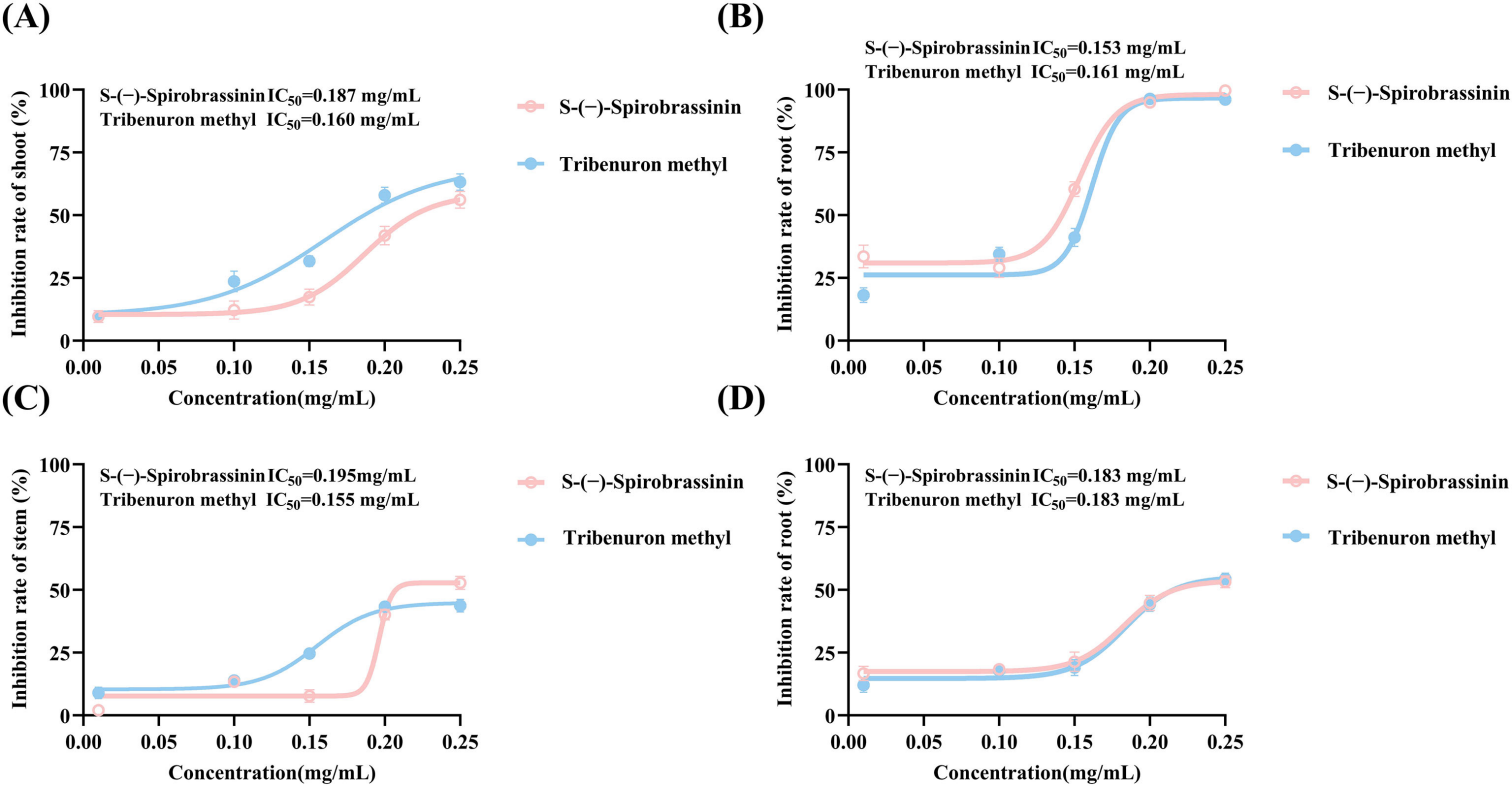
Dose dependent inhibitory effects of S-(-)-Spirobrassinin on *P. miliaceum* and *A. retroflexus*. **(A)** Inhibition of shoot length in *P. miliaceum* by S-(-)-Spirobrassinin. **(B)** Inhibition of root length in *P. miliaceum* by S-(-)-Spirobrassinin. **(C)** Inhibition of stem length in *A. retroflexus* by S-(-)-Spirobrassinin. **(D)** Inhibition of root length in *A. retroflexus* by S-(-)-Spirobrassinin. In all panels, the pink curve represents the fitted dose response relationship for S-(-)-Spirobrassinin, while the blue curve represents the corresponding fit for the positive control herbicide tribenuron methyl.

To clarify the dose-response relationship of S-(-)-Spirobrassinin, concentration-gradient experiments and IC_50_ calculations were conducted on the gramineous weed *P. miliaceum* and the broadleaf weed *A. retroflexus*. The results indicated that S-(-)-Spirobrassinin exhibited a lower IC_50_ value of 0.153 mg/mL for root inhibition in *P. miliaceum* and an IC_50_ of 0.187 mg/mL for shoot inhibition, outperforming the positive control herbicide tribenuron-methyl (**Figures 2A, B**), which suggests that the roots of *P. miliaceum* are more sensitive to S-(-)-Spirobrassinin. For *A. retroflexus*, tribenuron-methyl showed a smaller IC_50_ (0.155 mg/mL) for stem length inhibition compared to S-(-)-Spirobrassinin, while S-(-)-Spirobrassinin had an IC_50_ of 0.183 mg/mL for root length (**Figures 2C, D**). These findings collectively demonstrate that S-(-)-Spirobrassinin possesses higher activity against gramineous weeds than against broadleaf weeds.

### 3.3 Amplicon sequencing data analysis

To investigate the impact of S-(-)-Spirobrassinin on soil microorganisms, with biochar adsorptionserving as a positive control, the *ITS1* region of fungi and the *16S rRNA* region of bacteria in the soil were amplified and subjected to high-throughput sequencing. As shown in **Supplementary Table 1**, sequencing of the control soil yielded 91,350 *ITS* reads, of which 38,165were high-quality sequences, representing an effective rate of 41.8%. The biochar-treated soilgenerated 90,719 reads, with 38,750 high-quality sequences. The S-(-)-Spirobrassinin-treated soil produced 93,788 reads, with 34,324 high-quality sequences. The soil treated with both biochar and S-(-)-Spirobrassinin yielded 89,017 reads, of which 34,299 were high-quality sequences. From **Supplementary Table 2**, *16S rRNA* sequencing of the control soil generated 93,090 reads. After denoising and merging, 70,227 high-quality sequences were obtained, corresponding to an effective rate of 75.4%. The biochar-treated soil produced 91,691 reads, with 59,926 high-quality sequences. The S-(-)-Spirobrassinin treated soil yielded 91,412 reads, with 34,824 high-quality sequences. The soil treated with both biochar and S-(-)-Spirobrassinin generated 92,788 reads, resulting in 68,873 high-quality sequences. Therefore, the obtained sequencing depth was adequate for subsequent analysis.

### 3.4 Effects of S-(-)-Spirobrassinin on soil microorganisms at the phylum level

**Figure 3:**
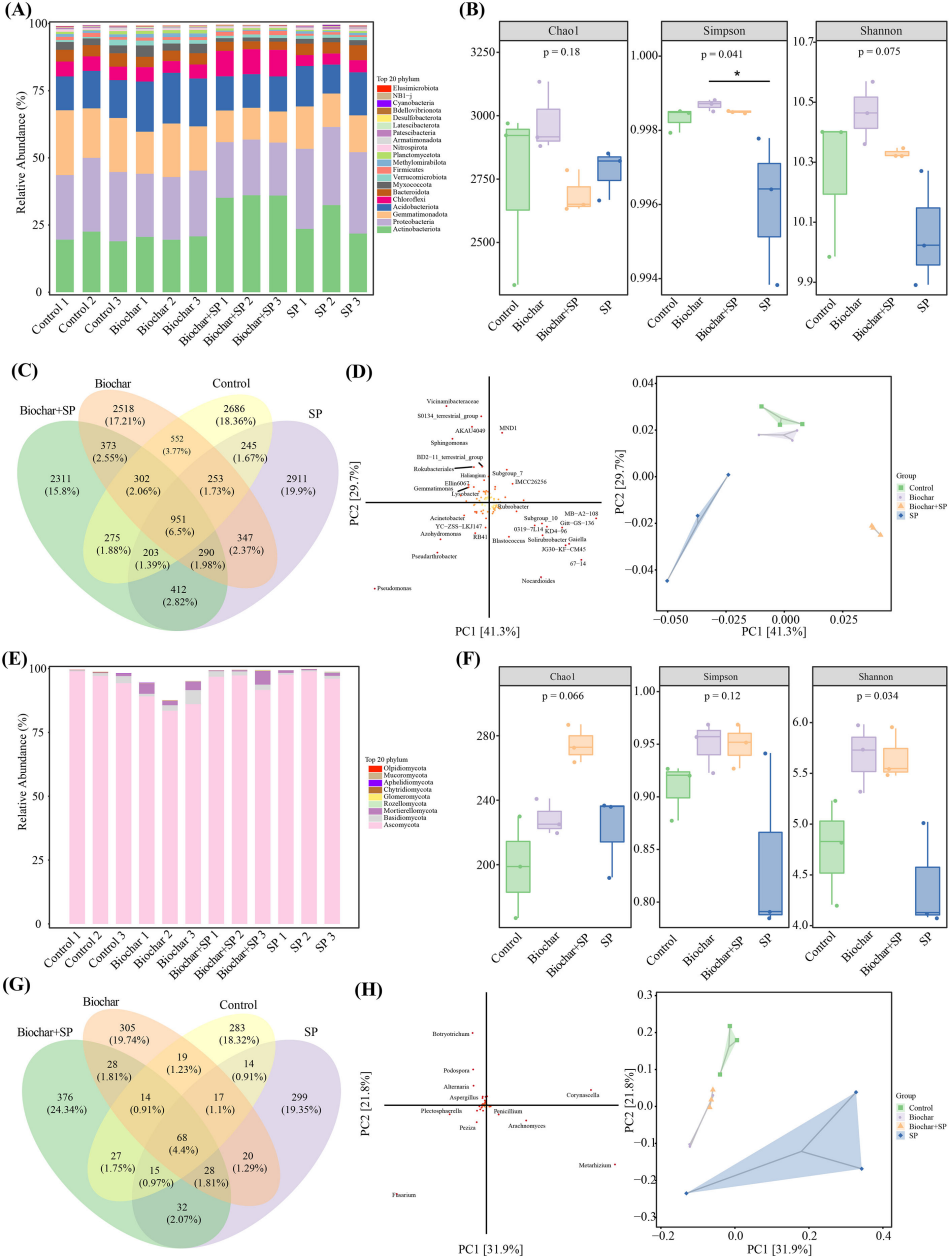
The effects of S-(-)-Spirobrassinin on soil microorganisms. **(A)** Bacterial community at the phylum level; **(B)** Bacterial alpha diversity; **(C)** Venn diagram of bacterial OTUs; **(D)** Bacterial beta diversity; **(E)** Fungal community at the phylum level; **(F)** Fungal alpha diversity; **(G)** Venn diagram of fungal OTUs; **(H)** Fungal beta diversity.

To investigate the effects of S-(-)-Spirobrassinin on soil microorganisms, sequencing analysis revealed that Proteobacteria, Actinobacteriota, Gemmatimonadota, and Acidobacteriota were the dominant bacterial phyla. The addition of S-(-)-Spirobrassinin resulted in the most pronounced increase in the relative abundances of Proteobacteria and Actinobacteriota compared to the control and biochar treated, while the relative abundance of Gemmatimonadota decreased. The co-addition of biochar and S-(-)-Spirobrassinin increased the relative abundance of Gemmatimonadota but reduced the abundance of Actinobacteriota in the soil (**Figure 3A**). At the fungal phylum level, the dominant phyla were Ascomycota, Basidiomycota, and Mortierellomycota. Compared to the control, the addition of biochar and S-(-)-Spirobrassinin significantly increased the relative abundances of Mortierellomycota and Basidiomycota. Furthermore, the addition of biochar alone significantly reduced the relative abundance of Ascomycota (**Figure 3E**).

### 3.5 Effects of S-(-)-Spirobrassinin on soil microbial community α-diversity

The α-diversity of soil bacteria was analyzed after 14 days of S-(-)-Spirobrassinin treatment. Results indicated that the addition of S-(-)-Spirobrassinin to the soil significantly reduced both the Simpson index and bacterial richness compared to the corresponding control, biochar treated, and the combined treatment of S-(-)-Spirobrassinin with biochar. While the inclusion of biochar mitigated the impact of S-(-)-Spirobrassinin on the Shannon index of soil bacteria, it did not alleviate its negative effect on the Simpson index (**Figure 3B**). The α-diversity indices of soil fungi were also significantly affected by S-(-)-Spirobrassinin, showing a notable decrease in the Shannon index and a significant reduction in fungal richness. However, the adsorptive capacity of biochar lessened the impact of S-(-)-Spirobrassinin on fungal richness in the soil (**Figure 3F**).

### 3.6 Principal component analysis of soil microbial communities under S-(-)-Spirobrassinin treatment

To illustrate the impact of S-(-)-Spirobrassinin on soil bacterial and fungal communities, principal coordinate analysis and cluster analysis were performed at the OTU level. **Figure 3D** clearly distinguishes the community structures of both bacteria and fungi under the different treatments. Specifically, S-(-)-Spirobrassinin altered the soil bacterial community structure, while the addition of biochar made its community composition more similar to that of the control soil. However, such a shift was not observed for the fungal community. The bacterial community analysis, as presented in **Figures 3C**, revealed that the soil treated solely with S-(-)-Spirobrassinin yielded the highest number of OTUs, totaling 2911. In contrast, the combined application of S-(-)-Spirobrassinin with biochar resulted in the lowest OTU count, which was 2311. This observation suggested that a portion of the bacterial community may have been adsorbed onto the biochar surface. Analysis of the fungal community in **Figure 3G** demonstrated a distinct enrichment effect associated with biochar. The addition of biochar alone led to the enrichment of 305 fungal OTUs. Notably, when the biochar was loaded with S-(-)-Spirobrassinin, the number of enriched fungal OTUs increased to 376. This finding indicated that the S-(-)-Spirobrassinin-biochar composite effectively enhanced the richness of the soil fungal community. S-(-)-Spirobrassinin application reduced fungal abundance in the soil. However, when combined with biochar, the fungal abundance was comparable to that in the biochar-only treatment and clustered closely with it in the community analysis (**Figure 3H**).

### 3.7 Degradation period of S-(-)-Spirobrassinin in soil

**Figure 4:**
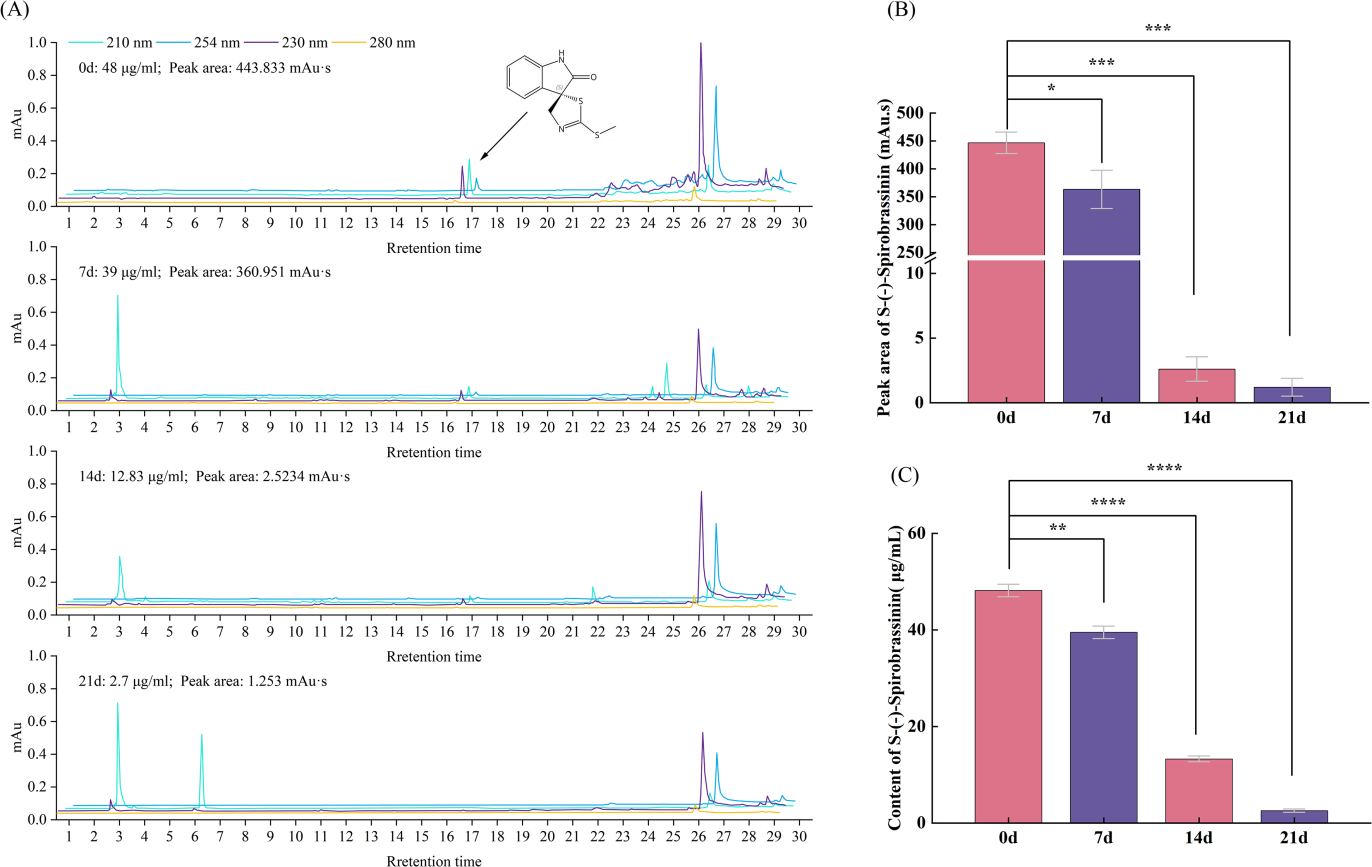
Degradation period of S-(-)-Spirobrassinin in soil. *p<0.05, **p<0.01, ***p<0.001, ****p<0.0001. **(A)** Chromatogram of S-(-)-Spirobrassinin degradation cycle; **(B)** Peak area of S-(-)-Spirobrassinin in soil at different times; **(C)** Content of S-(-)-Spirobrassinin in soil at different times.

A standard curve was established based on the peak area and the mass concentration of S-(-)-Spirobrassinin using the standard compound. As shown in **Figures 4A**, the standard compound exhibited a retention time of 16.704 min in the liquid chromatography analysis. The results indicated that S-(-)-Spirobrassinin is readily degradable in soil. Seven days after application, the compound was detected by liquid chromatography, and the peak area was converted to concentration, yielding a concentration of 39 μg/mL. After 14 days, the concentration decreased to 12.83 μg/mL, and after 21 days, it further declined to 2.7 μg/mL (**Figures 4B, C**).

### 3.8 Mean square displacement analysis

**Figure 5:**
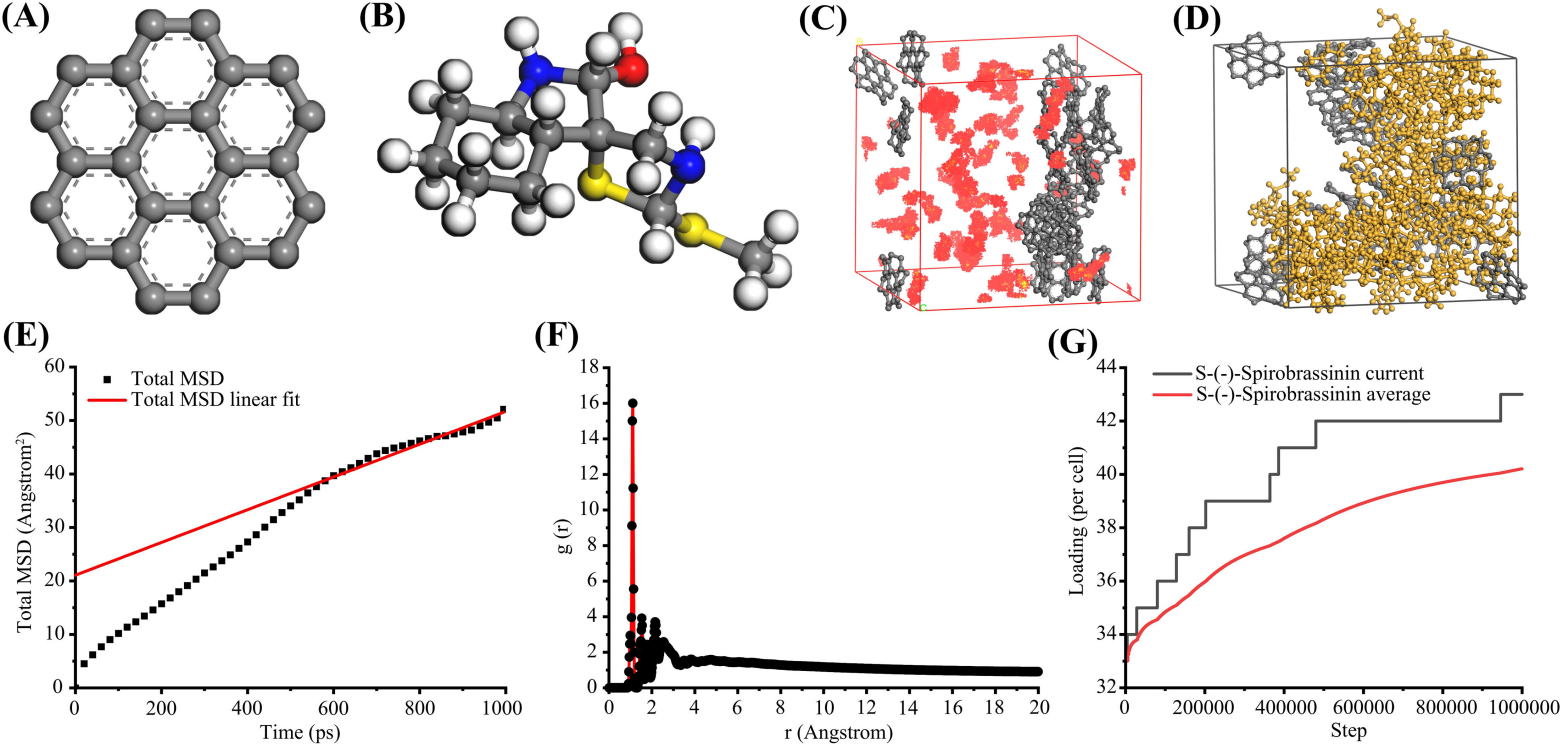
Molecular dynamics simulation of S-(-)-Spirobrassinin adsorption on biochar. **(A)** Seven-ring graphite sheet unit; **(B)** Three-dimensional structure of S-(-)-Spirobrassinin; **(C)** Simulated model of biochar material; **(D)** Simulation of S-(-)-Spirobrassinin adsorption on biochar; **(E)** Mean square displacement of S-(-)-Spirobrassinin in biochar; **(F)** Interaction between S-(-)-Spirobrassinin and atoms on the biochar surface; **(G)** Adsorption capacity of biochar for S-(-)-Spirobrassinin.

To better approximate the complex microstructure and chemical environment within biochar, we employed a graphene-like fragment consisting of seven aromatic rings as the fundamental structural unit (**Figure 5A**). Using this, we constructed a porous carbon material model and systematically investigated its adsorption behavior and mechanisms toward S-(-)-Spirobrassinin(**Figures 5B–D**). Based on the MSD data, the influence of biochar on the diffusion behavior of S-(-)-Spirobrassinin was analyzed. Within the simulated duration of 1 ns, the MSD of S-(-)-Spirobrassinin exhibited an approximately linear increase over time, rising from an initial value of 0 Å² to 52.11 Å² at 1000 ps. This indicates that S-(-)-Spirobrassinin displayed typical normal diffusion characteristics within the pores of the biochar. Combined with the strong adsorption site observed in the previous RDF analysis and the multilayer adsorption structure, it can be inferred that a transient adsorption-desorption dynamic equilibrium exists for S-(-)-Spirobrassinin on the biochar surface. Overall, the diffusion behavior is jointly regulated by the pore morphology and adsorption sites, exhibiting a confined yet sustained diffusion process (**Figure 5E**).

### 3.9 Analysis of radial distribution function

Based on the RDF data, the adsorption and diffusion behaviors between biochar and S-(-)-Spirobrassinin were analyzed. The results indicated the presence of a distinct adsorption structure near the biochar surface. The first adsorption layer was located at approximately 0.93 Å, while the strongest adsorption peak appeared at about 1.11 Å with a g(r) value as high as 16.01. This suggests that this position represents the most stable adsorption site for S-(-)-Spirobrassinin on the biochar surface, likely associated with strong interaction sites such as surface defects, functional groups, or micropore entrances. Subsequent multiple secondary peaks observed within the range of 1.5–3.0 Å further confirmed the characteristics of multilayer adsorption or ordered arrangement within the pore channels. Inr > 3.0 Å region, the g(r) values gradually decayed and approached 1.0, yet exhibited slight oscillations. This indicates that the pore structure imposes certain constraints on the diffusion behavior of S-(-)-Spirobrassinin (**Figure 5F**).

### 3.10 Analysis of sorption loading

Analysis of the Sorption Loading curve revealed that the adsorption rate was relatively slow during the 0-200,000 fs stage. As the simulation progressed, the adsorption quantity gradually increased and eventually stabilized, indicating that the system reached an adsorption-desorption equilibrium. The stabilized adsorption value reflects the maximum adsorption capacity of graphite for S-(-)-Spirobrassinin, with a final steady-state adsorption quantity of approximately 30–48 molecules per simulation box. The slope of the curve during the ascending phase corresponds to the adsorption rate. A steeper slope in the initial stage suggests a faster diffusion and adsorption process of S-(-)-Spirobrassinin molecules on the graphite surface. This may be attributed to the high specific surface area of graphite or strong initial interactions between S-(-)-Spirobrassinin and the graphite substrate. The subsequent decrease in slope indicates that adsorption sites were gradually occupied, leading to a slowdown in the adsorption rate (**Figure 5G**).

### 3.11 Analysis of isosteric heats of adsorption and specific surface area

As shown in **Table 1**, the average adsorption energy intensity is 25.185 kcal/mol, representing the mean energy strength for the adsorption of S-(-)-Spirobrassinin on the graphite surface. An adsorption heat around 25 kcal/mol falls within the transitional range between chemical and physical adsorption. The results indicate a strong interaction between S-(-)-Spirobrassinin and graphite, which may involve π-π stacking and electrostatic interactions. The minimum adsorption energy intensity of 6.092 kcal/mol corresponds to weak adsorption sites within the system, likely originating from defects on the graphite surface or regions where intermolecular repulsion occurs. Adsorption at such sites is relatively unstable, making S-(-)-Spirobrassinin molecules prone to desorption. The maximum adsorption energy intensity of 44.292 kcal/mol corresponds to strong adsorption sites, which may be unsaturated carbon atoms at the edges of graphene layers. At these sites, S-(-)-Spirobrassinin can form more stable adsorption configurations. The significantly higher adsorption heat compared to the average value demonstrates the heterogeneity of adsorption sites on the graphite surface, reflecting substantial energy differences among them. We further calculated the specific surface area (SSA) of the biochar model. After geometric and mass normalization, the SSA was determined to be approximately 268 m²/g.

**Table 1:** Simulated adsorption properties and structural parameters of biochar for S-(-)-Spirobrassinin.

Adsorption property	Level	Value
Isosteric heats(kcal/mol)	Maximum	44.292
	Average	25.185
	Minimum	6.092
Sorption loading(molecules/box)	Maximum	43
	Average	42.208
	Minimum	33
Specific surface area(m²/g)		268

## 4 Discussion

Although isolated from broccoli, the herbicidal selectivity of S-(-)-Spirobrassinin remained unclear. To evaluate its potential for future application, this study assessed its herbicidal selectivity and crop safety by screening four crop and five weed species. The results revealed that *S. italica* exhibited tolerance to S-(-)-Spirobrassinin. Graminaceous weeds were more strongly inhibited than broadleaf weeds. Additionally, weed seed size did not appear to be a limiting factor for the allelopathic action of S-(-)-Spirobrassinin. Dose response assays further confirmed its higher efficacy against graminaceous weeds. S-(-)-Spirobrassinin strongly inhibited root growth in *P. miliaceum*, outperforming the conventional herbicide tribenuron methyl. In contrast, its effect on the broadleaf weed *A. retroflexu*s was weaker, a difference potentially attributable to divergent uptake or metabolic pathways between plant types (Torra et al., 2024). Future studies should examine how S-(-)-Spirobrassinin inhibits graminaceous weeds.

The safety of herbicides to soil microorganisms represents a critical environmental and ecological concern, given the pivotal role soil microbes play in maintaining ecosystem function and soil health (Parven et al., 2025; Lv et al., 2025; Shultana et al., 2025). Alterations in soil microbial structure and diversity can directly or indirectly influence plant growth and development (Nie et al., 2025). As S-(-)-Spirobrassinin shows promise as a lead compound for botanical herbicides, its safety profile must be thoroughly considered. Although it is a natural secondary metabolite from broccoli and is presumably biodegradable in the environment, its application as a pesticide would inevitably impact soil microbes. Our findings indicate that S-(-)-Spirobrassinin significantly increased the relative abundance of Proteobacteria and Actinobacteriota in the soil. Being a nitrogen-containing organic compound, S-(-)-Spirobrassinin can be decomposed and metabolized by microorganisms like those in Actinobacteriota, which are involved in organic matter decomposition and the nitrogen cycle. This microbial metabolism supports the inherent biodegradability and reduced environmental persistence of S-(-)-Spirobrassinin. The increase in Proteobacteria abundance may represent a self-regulatory response of the soil microbial community to adapt to the presence of S-(-)-Spirobrassinin (Zhao and Wang, 2025). As S-(-)-Spirobrassinin is acidic, its introduction lowered soil pH. Studies have shown a positive correlation between soil pH and the abundance of Actinobacteriota (Baćmaga et al., 2025). Research suggests that increased abundances of Proteobacteria and Actinobacteriota can indirectly promote plant growth (Yanxia et al., 2025). Furthermore, the combined application of biochar and S-(-)-Spirobrassinin promoted the population size of the fungal community in the soil. On one hand, this indicates that the two can be used in combination with relatively limited impact on the soil microbial community. On the other hand, the readily mineralizable carbon in biochar likely served as a microbial carbon source, potentially stimulating the growth of saprophytic fungi (Dai et al., 2018). This may have enhanced their self-propagation and competitive ability, leading to an overall decline in community diversity.

Biochar, as a non-polar adsorbent, holds broad application prospects in agriculture as a sustained-release carrier (Murtaza et al., 2025; Lin et al., 2025). Given these advantages, we investigated its interaction with S-(-)-Spirobrassinin. Molecular dynamics simulations revealed that biochar exhibits strong adsorption affinity for S-(-)-Spirobrassinin. The application of microencapsulation technology significantly enhanced the herbicidal activity of trifluralin against target weeds while reducing its toxicity to non-target crops (Cao et al., 2019). Polyurea microcapsules have been used to encapsulate pretilachlor, achieving effective control of barnyard grass with minimized toxicity to rice, which aligns with the goals of reduced pesticide use and enhanced efficiency (Chen et al., 2021). Similarly, multi-layered sodium alginate/polysaccharide composites have served as a controlled-release matrix for glufosinate, demonstrating excellent sustained-release performance and biosafety (Li et al., 2024). Furthermore, a sodium alginate-urea-fluroxypyr hydrogel system has been developed to enable the synergistic slow release of both herbicide and fertilizer (Chen et al., 2025).

Our results exhibit strong adsorption affinity for S-(-)-Spirobrassinin, which are poorly water-soluble and have low polarity. In this study, molecular dynamics simulations were employed to systematically investigate the adsorption and diffusion behaviors of S-(-)-Spirobrassinin on biochar and to evaluate its potential as a slow-release carrier from a mechanistic perspective. RDF analysis revealed a significant peak for the herbicide molecule on the carbon surface at approximately 1.11 Å (g(r) ≈ 16.01), indicating the presence of high energy adsorption sites. These likely involve interactions such as hydrogen bonding or electrostatic forces with functional groups at micropore entrances, edge defects, or on the surface. Biochar interacts with aromatic herbicides via π-π stacking, suggesting that carbon materials can achieve strong immobilization of organic pollutants through multiple non-covalent interactions (Chang-Chien, 2025). The high specific surface area further ensures sufficient adsorption capacity, providing a structural basis for drug loading. MSD analysis indicated a diffusion coefficient of approximately 7.6 × 10^-6^ cm²/s for the herbicide within the carbon pores. This restricted diffusion is primarily attributed to the combined effects of spatial hindrance from micropores and surface interactions, which directly prolongs the molecule’s residence time within the material, facilitating slow release.

The adsorption curve showed that the adsorption process on the carbon material followed an “initial slow-mid fast-late equilibrium” pattern, consistent with a diffusion-controlled pore-filling mechanism (Samokhvalov and Tatarchuk, 2010). Similarly, the desorption process typically exhibits an initial rapid phase for weakly bound molecules, followed by a slower release of molecules from strong binding sites (Delle Site, 2001). This multi-stage release behavior holds potential for achieving a “quick onset-sustained maintenance” release profile in practical applications, aligning with the design requirements for agricultural slow-release formulations (Luhach et al., 2025).

The 1 ns simulation time in this study efficiently captured the dominant interaction modes between S-(-)-Spirobrassinin and the carbon surface, such as π-π stacking and hydrogen bonding, which are typically established on a nanosecond timescale. Although this timeframe is insufficient to fully characterize long-term diffusion and desorption kinetics, it is adequate for identifying the primary binding configurations and interaction energies that underpin the observed multi-stage release behavior. It should be noted that our molecular model represents a simplified, homogeneous graphitic surface. Real biochar contains a distribution of pore sizes and heterogeneous functional groups (Qian and Chen, 2013), which may influence adsorption kinetics. Although the simulations were conducted under idealized conditions, such as soil moisture, pH, ionic strength, and organic matter content can influence the desorption kinetics of the herbicide. For instance, acidic conditions might enhance electrostatic attraction between positively charged herbicides and negatively charged carbon surfaces, delaying release (Xie et al., 2025), while dissolved organic matter in soil could compete for adsorption sites, potentially accelerating release (Kaur et al., 2025). Future simulations will incorporate these complexities to build more realistic structure-property relationships and guide the rational design of biochar with tailored release properties.

Compared to traditional polymer carriers, biochar offers advantages such as lower cost, better environmental compatibility, and higher adsorption capacity (Yasim-Anuar et al., 2022). However, its release profile might be more gradual, making it potentially more suitable for scenarios requiring long-term, stable release (Zhang et al., 2021). Furthermore, the pore structure and surface chemistry of biochar can be tuned through feedstock selection and pyrolysis conditions, enabling a degree of “designability” in its release behavior.

In summary, this study systematically evaluated the potential of S-(-)-Spirobrassinin as a botanical herbicide lead compound and the feasibility of biochar as its carrier, spanning from field selectivity to molecular mechanisms. The compound demonstrated promising selectivity by strongly inhibiting graminaceous weeds while showing minimal effects on *S. italica*, identifying a safe crop for its potential application. From an environmental safety perspective, S-(-)-Spirobrassinin, especially when combined with biochar, exhibited a relatively limited impact on soil microbial community structure. It was metabolized by specific bacterial phyla and did not reduce fungal diversity, indicating favorable environmental compatibility. At the molecular level, the strong adsorption capacity, restricted diffusion, and multi-stage kinetics of S-(-)-Spirobrassinin on biochar form the physicochemical foundation for controlled release. Collectively, these findings provide an integrated theoretical basis.

## Data Availability

The raw data supporting the conclusions of this article will be made available by the authors, without undue reservation.
