# Supplementary material for: Integrated assessment of selectivity, soil behavior, and biochar-mediated release for the botanical herbicide precursor S-(-)-Spirobrassinin
Source: Front Plant Sci. 2026 Mar 6;17:1787286. doi: 10.3389/fpls.2026.1787286 (PMC13012011; doi:10.3389/fpls.2026.1787286)
Supplement: Supplementary file 1 [file DataSheet1.pdf]

Supplementary Material

Supplementary Table 1. Preprocessing of *ITS* amplification sequencing

Treatment	Total sequence number	Filtered sequence number	Denoised sequence number	Menged sequence number	Valid sequence number
Control	90 710	75 182	74 747	73 145	69 225
	90 366	73 443	72 903	70 671	67 208
	98 194	80 500	79 938	77 860	74 249
Biochar	91 541	62 196	61 646	58 553	54 747
	92 435	65 970	65 173	62 192	60 014
	91 097	72 581	72 035	69 813	65 018
Biochar+ S-(-)-Spirobrassinin	95 685	77 718	76 923	73 183	70 572
	91 908	76 799	75 923	73 350	69 036
	90 773	75 958	75 208	72 468	67 011
S-(-)-Spirobrassinin	92 519	72 828	72 296	70 375	68 389
	92 298	77 753	77 193	73 553	63 523
	89 419	68 906	68 312	65 831	63 855

Supplementary Table 2. Preprocessing of *16S rRNA* amplification sequencing

Treatment	Total sequence number	Filtered sequence number	Denoised sequence number	Menged sequence number	Valid sequence number
Control	87 478	73 708	69 725	40 986	37 774
	92 309	77 685	72 810	39 999	36 687
	94 263	79 385	74 341	41 608	38 217
Biochar	93 231	79 126	74 371	43 927	40 858
	88 594	74 723	70 375	40 103	36 985
	90 333	76 969	71 906	39 781	36 564
Biochar + S-(-)-Spirobrassinin	87 625	73 840	68 454	35 102	32 612
	89 906	76 143	70 736	36 451	34 016
	89 521	75 216	69 862	34 379	31 765
S-(-)-Spirobrassinin	91 539	77 355	72 007	35 811	31 943
	94 298	78 987	74 051	40 738	36 461
	95 528	80 971	75 304	37 412	33 645

Supplementary Table 3. Analytical method performance for the determination of S-(-)-Spirobrassinin in soil

Parameter	Value
Limit of detection (LOD)	48.8 ng/g
Limit of quantification (LOQ)	162.7 ng/g
Absolute recovery	81.3% (RSD = 9.9%)
Calibration linearity (R^2)	> 0.999
